# A Wearable and Real-Time Pulse Wave Monitoring System Based on a Flexible Compound Sensor

**DOI:** 10.3390/bios12020133

**Published:** 2022-02-20

**Authors:** Xiaoxiao Kang, Jun Zhang, Zheming Shao, Guotai Wang, Xingguang Geng, Yitao Zhang, Haiying Zhang

**Affiliations:** 1 Institute of Microelectronics of Chinese Academy of Sciences, Beijing 100029, China; kangxiaoxiao@ime.ac.cn (X.K.); zhangjun@ime.ac.cn (J.Z.); shaozheming@ime.ac.cn (Z.S.); wangguotai@ime.ac.cn (G.W.); gengxingguang@ime.ac.cn (X.G.); zhangyitao@ime.ac.cn (Y.Z.); 2 University of Chinese Academy of Sciences, Beijing 100049, China; 3 Beijing Key Laboratory for Next Generation RF Communication Chip Technology, Beijing 100029, China

**Keywords:** wearable, flexible compound sensor, wrist pulse signal, real-time monitoring, the varying trend of pulse wave peak, best pulse wave positioning

## Abstract

Continuous monitoring of pulse waves plays a significant role in reflecting physical conditions and disease diagnosis. However, the current collection equipment cannot simultaneously achieve wearable and continuous monitoring under varying pressure and provide personalized pulse wave monitoring targeted different human bodies. To solve the above problems, this paper proposed a novel wearable and real-time pulse wave monitoring system based on a novel flexible compound sensor. Firstly, a custom-packaged pressure sensor, a signal stabilization structure, and a micro pressurization system make up the flexible compound sensor to complete the stable acquisition of pulse wave signals under continuously varying pressure. Secondly, a real-time algorithm completes the analysis of the trend of the pulse wave peak, which can quickly and accurately locate the best pulse wave for different individuals. Finally, the experimental results show that the wearable system can both realize continuous monitoring and reflecting trend differences and quickly locate the best pulse wave for different individuals with the 95% accuracy. The weight of the whole system is only 52.775 g, the working current is 46 mA, and the power consumption is 160 mW. Its small size and low power consumption meet wearable and portable scenarios, which has significant research value and commercialization prospects.

## 1. Introduction

Pulse wave diagnosis has a glorious history both in China and India. Studies have shown that pulse waves are the intuitive reflection of the state of the internal heart and blood vessels [1,2,3,4,5]. It has been proven in modern medicine to be able to predict and reflect a variety of diseases, such as cardiovascular disease [6] and diabetes [7]. The intensity of the radial artery pulse wave is considered an indicator for the diagnosis of many diseases. Moreover, its characteristics of non-invasiveness, non-radiation, and relatively simple processes have been widely accepted and concerned [8].

Wearable and continuous monitoring of physiological signals such as pulse wave has gradually become a research hotspot [9,10,11,12]. Some studies have used wearable sensors to monitor arterial waveforms such as photoplethysmography (PPG) signals, combined with novel machine learning algorithms, to establish new ways to advance the progress of physiological health monitoring, and made remarkable progress [9,11]. Pulse wave diagnosis can obtain the varying trend of pulse wave peak by applying different levels of static pressure to the radial artery, which can reflect the current physical state of the observer [10,12]. Since each person has different static pressure ranges, it is necessary to measure the pressure of the radial artery to obtain an individualized varying trend of pulse wave peaks. Traditional pulse wave diagnosis mainly relies on the experience of Traditional Chinese Medicine (TCM), and the diagnostic criteria vary from person to person. Objective and quantitative diagnostic equipment has been a research hotspot for decades [13].

There are some problems with the pulse wave sensor. The main categories of pulse wave pressure sensors include photoelectric sensors [14,15,16,17], piezoresistive sensors [18,19], ultrasonic sensors [20], and pressure sensors [21]. Photoelectric sensors, such as PPG sensors, which have made some notable progress [14,16,17], are susceptible to light interference from external sources, and cannot measure the trend of pulse wave pressure under continuously varying pressure. The sensitivity of the piezoresistive sensor [18] is inversely proportional to the pressure range, so when the applied pressure is much larger than the pulse wave, it is difficult to detect the weak pulse wave signal and obtain high-quality data. The pressure sensor converts the mechanical pressure into an electrical signal, which imitates the doctor’s tactile sense of pulse in practice, which is considered to be a better choice. The flexible pressure sensor based on Polyvinylidene fluoride (PVDF) piezoelectric sensor can be used for wearable pulse measurement, such as [22], it is still unable to obtain the pulse wave and applied pressure information under continuously varying pressure during the test.

There are also some defects in the existing pulse wave acquisition equipment. In our previous work [1,2], we proposed a new type of pulse wave acquisition device that can automatically pressurize the radial artery in sections, but the device is large in size, poor in portability, and high in power consumption, which cannot meet the needs of continuous pulse wave collection and real-time monitoring. This problem also exists in other jobs [22]. There are also some wearable pulse wave acquisition devices that have been verified in principle, but there are still problems such as the inability to perform real-time calculations or to apply continuously changing pressure [23,24,25]. Building a wearable pulse wave monitoring system to obtain simultaneously pulse wave and pressure information under continuously varying pressure is one of the urgent problems to be solved.

To solve the above problems, this paper proposed a wearable and real-time monitoring system based on a flexible compound sensor, which can simultaneously obtain pulse wave and pressure information under continuously varying pressure. Firstly, the flexible compound sensor includes a custom-packaged pulse pressure sensor, a signal stabilization structure, and a micro pressurization system. While applying continuously changing static pressure to the radial artery, it achieves stable acquisition of pulse wave signals. Then, a conditioning circuit was designed for pulse signal processing and an algorithm was developed to obtain the varying trend of the pulse wave peak under varying pressure in real-time, so as to calculate the characteristic parameters of the pulse wave. Finally, experiments are carried out to verify the accuracy, repeatability, and effectiveness of the flexible compound sensor. The results show that the compound sensor has good accuracy and repeatability, and the system can not only obtain the pulse wave under continuously varying pressure, but also analyze the varying trend of the pulse wave peak with pressure in real-time, which can be used by different people and quickly locate the strongest point of pulse wave.

## 2. Materials and Methods

### 2.1. System Overview

**Figure 1:**
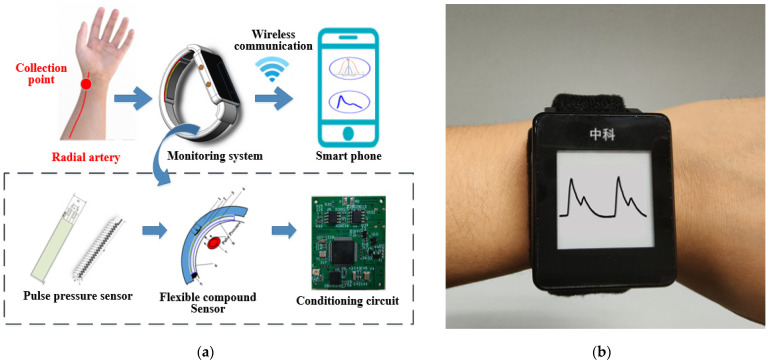
The proposed pulse wave monitoring system. (**a**) Schematic diagram of the monitoring system, based on a flexible compound sensor; (**b**) Photograph of the monitoring system.

The wearable and real-time pulse wave monitoring system proposed in this paper includes a flexible compound sensor that is worn on the human wrist to complete the pulse wave signal acquisition, a circuit structure to accomplish the signal processing and transmission, and a real-time algorithm to realize the calculation of the pulse wave under different static pressures. Specifically, the flexible compound sensor consists of three parts: a pulse wave pressure sensor to collect pulse wave signals, a signal stabilization structure customized to ensure signal quality, and a micro pressurization system to apply continuous varying pressure to the radial artery. The schematic diagram of the monitoring system is shown in Figure 1a and the photograph of the monitoring system is shown in Figure 1b.

Firstly, the pulse wave signal is collected by the flexible compound sensor under the continuously varying pressure, and then transmitted to the circuit, conveyed to the microprocessor for real-time algorithm processing to obtain information such as the varying trend of pulse wave peak. Simultaneously, it was formed feedback based on the calculation results to control the micro pressurization system. The original data and algorithm results can be displayed on the low-power ink screen that comes with the system. Furthermore, they can be transmitted to a PC or smartphone via wireless communication for real-time monitoring. The weight of the whole system is only 52.775 g, the maximum working current is approximately 46 mA, and the power consumption of the whole machine is approximately 160 mW, which meets the requirements of wearable and portable scenarios.

### 2.2. Flexible Compound Sensor

**Figure 2:**
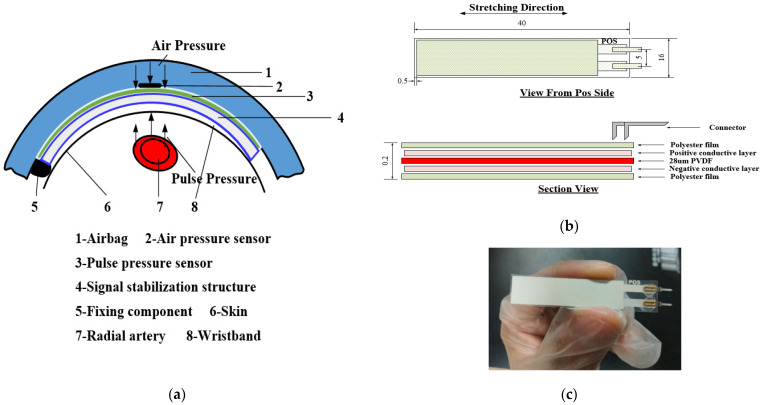
Flexible compound sensor: (**a**) Schematic diagram of the flexible compound sensor; (**b**) Schematic diagram of the package structure of the pulse pressure sensor; (**c**) Photograph of the pulse pressure sensor.

The flexible compound sensor is the key to the design of the entire wearable system that is designed to support the stable and effective collection of pulse waves under continuously varying pressure. In order to closely fit the human wrist to collect pulse waves, the flexible compound sensor consists of a pulse pressure sensor, a signal stabilization structure, and a customized micro pressurization system. A schematic diagram of the flexible compound sensor is shown in the Figure 2a. The solid line shows the specific component, and the broken line indicates the name of the specific component.

#### 2.2.1. Pulse Pressure Sensor

To obtain an effective pulse wave signal, we designed the pulse pressure sensor based on the PVDF piezoelectric sensor, which has high sensitivity and meets the frequency characteristics of the pulse signal. Its dynamic range completely covers the pulse beating range and can effectively cover the pulse wave collection site on the wrist. The characteristic table of the sensor is shown in Table 1.

To collect the pulse wave stably and effectively, considering the sensing, protection, and external connection, we customized the pulse pressure sensor into a five-layer structure. The schematic diagram of the package structure is shown in Figure 2b. From top to bottom, they are the top protective layer, the positive conductive layer, and the PVDF piezoelectric thin film sensor (28 μm), the negative conductive layer, and the bottom protective layer. The top and bottom protective layers are made of polyester film. On the one hand, it protects the PVDF film material and conductive layer inside the sensor from being damaged by moisture and mechanical friction. On the other hand, it can also provide mechanical strength for the pins. The positive and negative conductive layers are printed with silver ink on both sides of the film as electrodes on both sides of the film. When the PVDF piezoelectric sensor is deformed by force, polarized charges are generated on both sides of the film, and the positive charge side passes through the positive conductive layer. It is transferred to the positive pin, and the negative charge side is transferred to the negative pin through the negative conductive layer. The photograph of the pulse pressure sensor is shown in Figure 2c. The sensitive area of the sensor is determined by the PVDF piezoelectric sensor, which is 40 × 10 mm and can effectively cover the pulse wave collection site on the wrist. The distance between the boundary of the shape and the boundary of the sensitive surface is 0.5 mm. The connector uses a pin with a length of 10 mm to conduct the charge out.

Considering the design of the package, we try to fix the shape of the film itself and consider trying our best to reduce the interference caused by the space electromagnetic and the stability of the connector. Firstly, to prevent the fluctuation of the shape of the film itself from generating noise, we use packaging and pulse signal stabilization structure to limit the freedom of the film shape in the application. Secondly, the sensor lead is connected with a twisted pair when it is connected to the pin, which can not only transmit the signal of the PVDF piezoelectric sensor but also shield the external electromagnetic interference. Finally, to ensure the stability of the connector, in our research, the sensor is connected by a pin and the lead is connected by solder.

#### 2.2.2. Pulse Signal Stabilization Structure

**Figure 3:**
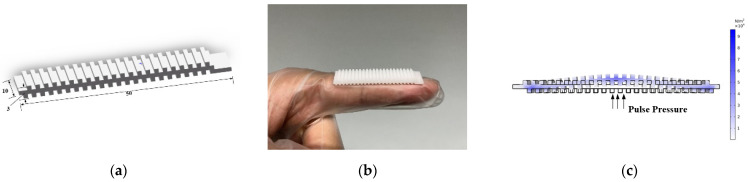
Signal stabilization structure: (**a**) Schematic diagram of the signal stabilization structure; (**b**) Photograph of the signal stabilization structure; (**c**) Schematic diagram of stress analysis of the signal stabilization structure.

To enhance the sensor sensitivity and effectively collect pulse waves, based on the above package structure, we have customized a signal stabilization structure using soft rubber (length: 50 mm, width: 10 mm, thickness: 3 mm, Shore hardness: A30, 3D printing), which is applied between the skin and the sensor. The schematic diagram of the signal stabilization structure is shown in Figure 3a. Soft rubber has good toughness and elasticity, high heat resistance, tear resistance, and soft texture, which can fit the wrist closely and increase the friction between the skin and the sensor so that the stability of the pulse signal is at the sensor position. The photograph of the signal stabilization structure is shown in Figure 3b. The zigzag shape design of the module can effectively transmit the deformation of the PVDF film. The schematic diagram of stress analysis of the signal stabilization structure is shown in 3c. The stress analysis of the signal stabilization structure shows that when the pulse wave pressure acts on the lower surface, it can be regarded as a rigid body in a steady state. After being transmitted to the upper surface, the pressure at the contact points on the upper surface can be increased to make the signal more stable and sensitive to capture by the PVDF sensor structure.

#### 2.2.3. Micro Pressurization System

To obtain the varying trend of the pulse wave peak of the pulse wave under continuously varying pressure, we designed a micro pressurization system to apply continuously varying pressure on the radial artery. The pressure range is from 0 mmHg to 180 mmHg, which is in line with the small size and low power consumption design to meet wearable and portable scenarios. The micro pressurization system mainly includes an integrated pump, air pressure sensor (MPS20N0040D-S), and inflatable wrist strap. To meet the needs of wearable and portable design, we customized an integrated air pump (thickness is only 6 mm and weight is only 2.48 g) that combines an air pump and a solenoid valve. The integrated pump is controlled by the microcontroller unit (MCU) to inflate the customized micro wristband, maintain the pressure, and deflate the pressure. The air pressure sensor has good repeatability and long-term stability, which is used to measure the air pressure in the micro pressurization system and feedback the current air pressure value to the MCU for further control.

**Figure 4:**
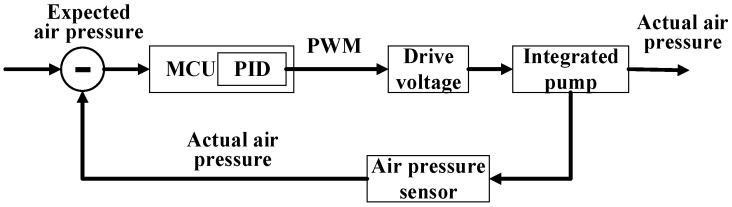
Flow chart of air pressure stabilization control.

To keep the air pressure stable, we propose an integrated air pump control method based on the Proportion Integration Differentiation (PID) algorithm. Flow chart of air pressure stabilization control is shown in Figure 4. The difference between the expected air pressure value and the actual air pressure value is used as the algorithm input. Through proportional and differential adjustment, the output feedback error drive voltage is superimposed on the original drive voltage. The MCU controls the driving voltage by changing the duty cycle of the Pulse Width Modulation (PWM) wave to change the pressing speed. To stabilize the air pressure, adjust the air pressure so that it stabilizes at the optimal air pressure value and fluctuates no more than 3%. According to the personalized inflation speed, the integrated air pump can be controlled by MCU to perform stable inflation and deflation; also, the air pump achieved to inflate to any pressure value and maintain it continually.

When the pulse wave is collected, the flexible compound sensor is pressed above the radial artery of the wrist, and the micro pressurization system applies a continuously changing static pressure to the radial artery. The force generated by the pulse is transmitted through the skin and the pulse signal stable structure. The sensitive area of the sensor captures the deformation, the sensor generates a polarized charge based on the piezoelectric effect, and then the charge is transferred to the pin, through the pre-amplifier circuit, to complete the collection of the pulse wave electrical signal.

### 2.3. Signal Processing

#### 2.3.1. Circuit Architecture

**Figure 5:**
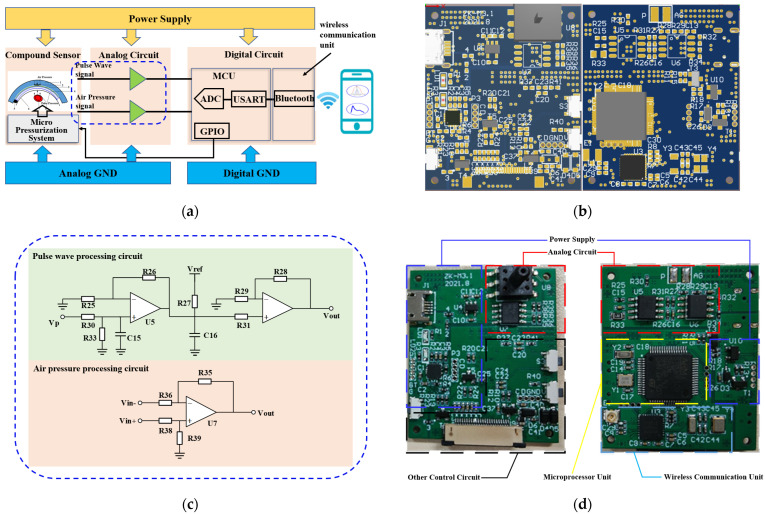
Schematic diagram of the circuit system: (**a**) Circuit system function diagram; (**b**) Detailed design in the blue dash frame in (**a**); (**c**) Printed circuit board layout view; (**d**) Photograph of the circuit system.

To meet the needs of a wearable and mobile monitoring system, the circuit architecture adopts low power consumption and miniaturization design, which mainly includes signal processing circuit, analog-digital converter (ADC) circuit, control circuit, and power supply module. Schematic diagram of circuit architecture is shown in Figure 5. The voltage supply is powered by a 4.2 V rechargeable lithium battery with a rated capacity of 270 mAh. The wrist skin is connected to the system ground through a wire, and the reference ground electrode is introduced on the skin to reduce the interference of space charge on the PVDF signal acquisition, which can effectively eliminate the 50 Hz power frequency signal and greatly simplifies the circuit.

In the signal acquisition process, firstly, the pulse pressure sensor transfers the polarized charge to the pre-charge amplifier circuit through the pins and leads. The high-impedance input can well capture the weak charge generated by the PVDF film. Then a preamplification circuit is a voltage amplifier with a voltage gain of 11 times. After that the signal is conveyed to the MCU to calculate the varying trend of the pulse wave peak in real-time and transmitted to the smartphone or PC via wireless communication. The signal sampling frequency is 250 Hz.

#### 2.3.2. Real-Time Calculation Algorithm

**Figure 6:**
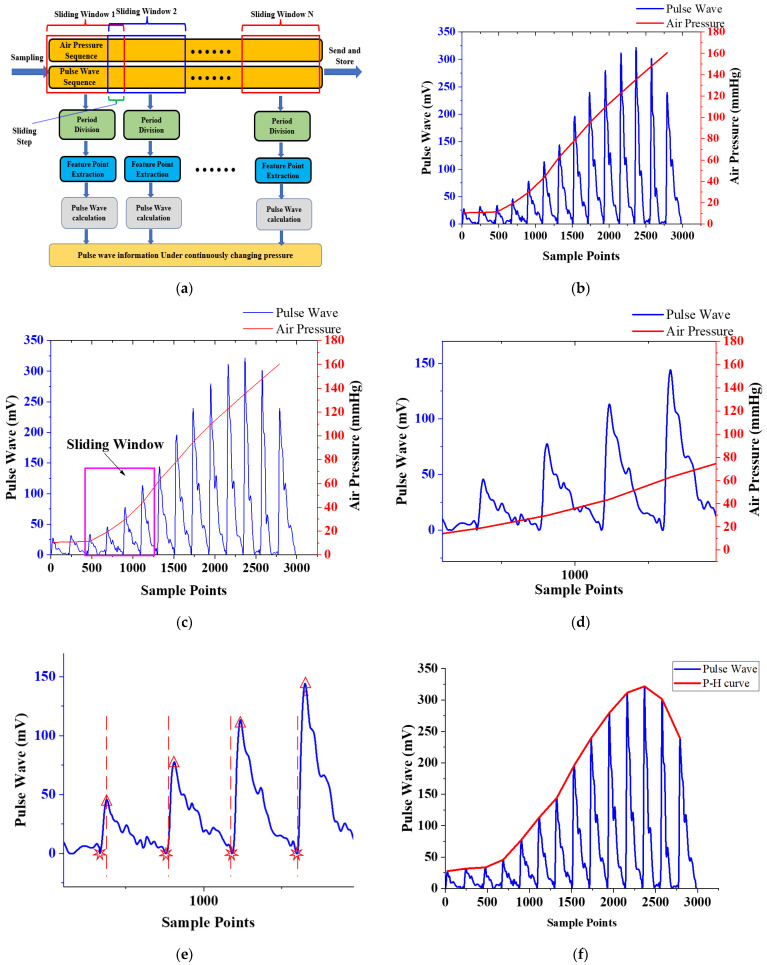
Real-time calculation algorithm: (**a**) Schematic diagram of the real-time algorithm diagram; (**b**) Filtered pulse wave signal and air pressure signal; (**c**) Schematic diagram of sliding window; (**d**) The signal in the sliding window in Figure 5c; (**e**) Location of the starting point of the pulse wave and period division; (**f**) Pulse wave and P-H curve.

To ensure the real-time performance of the calculation, we calculate the varying trend of pulse wave peak on the MCU. The calculation result can be output and displayed on the screen of the system in real-time, or transmitted to the PC or smartphone via wireless communication. Before this, the collected signal will be filtered to remove noise. We use a median filter to remove abnormal points in the sampling process, and an average filter and a low-pass filter with a cutoff frequency of 15 Hz to filter out the interference of baseline drift. The filtered pulse wave signal and air pressure signal is shown in Figure 6b.

To calculate the pulse wave parameters and the varying trend of pulse wave peaks in real-time, we proposed a calculation method based on a sliding window. The schematic diagram of the real-time algorithm is shown in Figure 6a. The schematic diagram of sliding window is shown in Figure 6c. The size of sliding window is 1024 sampling points, and the sliding step size is set to 250 sampling points. For the pulse wave signal in the window, shown in Figure 6d, firstly, locate the pulse wave starting point for real-time period division, shown in Figure 6e. Secondly, according to the position of the characteristic point, calculate the pulse wave peak, pulse rate, and other parameters. The pulse wave in the signal sequence is monitored to obtain the static pressure corresponding to the pulse wave with the strongest amplitude, to realize the accurate positioning and continuous monitoring of the best pulse wave of different individuals.

The dynamic pulse pressure curve generated by a sensor takes the peak value under static pressure as the horizontal axis, and the air pressure value of the pulse wave peak value as the vertical axis of the varying trend of pulse wave peak. It depicts the varying trend of the wrist pulse wave peak under continuously varying pressure, hereinafter referred to as the pressure-height (P-H) curve, shown in Figure 6f, which fully describes the depth information of the radial artery.

## 3. Experimental Results and Discussion

### 3.1. Test Device for Performance Test of the Flexible Compound Sensor

**Figure 7:**
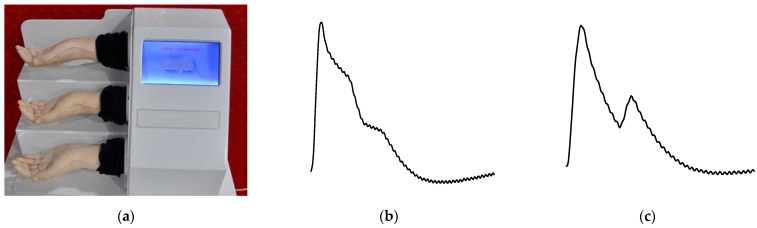

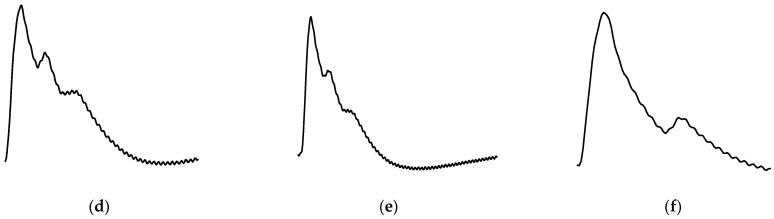
Test device for the flexible compound sensor. (**a**) Photograph of the test device; (**b**–**f**) the pulse generated by the test device called, respectively, Ping, Xian, Hua, Ji, and Chi.

To verify the reliability, consistency, and effectiveness of the flexible compound sensor, we employed a standard pulsation signal source, the MM-4 pulse simulator, shown in Figure 7a. The equipment based on bionic simulation and waveform synthesis methods, develops bionic hands and radial artery blood vessels with polymer material formulas, and uses stepping speed-regulating motors and special oil pumps to simulate the dilation and contraction of the human heart. It can output a variety of standard finger-sensing real pulse wave signals at the radial artery of the bionic hand. We can set the shape of the pulse wave independently. Figure 7 shows the pulse shapes called Ping, Xian, Hua, Ji, and Chi generated by the pulse simulator, which are considered as the five common pulse sharps in TCM.

### 3.2. Verification of Airtightness

**Figure 8:**
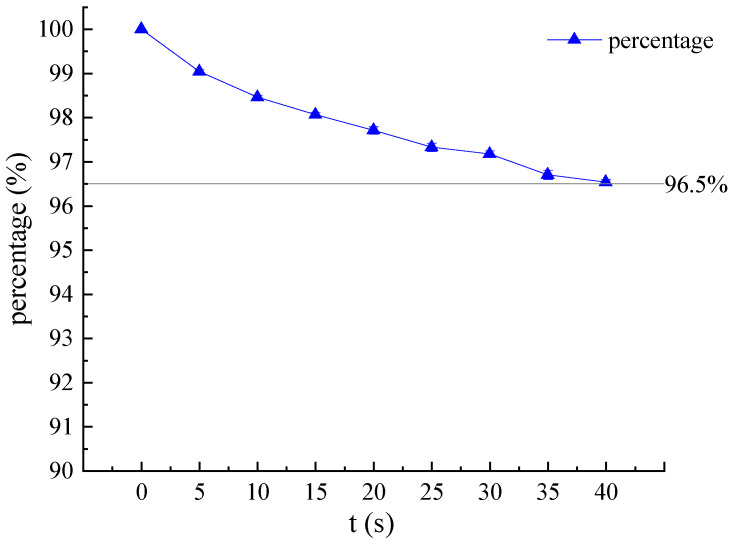
Air tightness of the flexible compound sensor under 150 mmHg airbag pressure for 40 s.

The micro pressurization system provides a continuously changing static pressure for the flexible compound sensor, which is an important part of the entire system. Initially, the airtightness test of the micro pressurization system in the flexible compound sensor is carried out. Firstly, wear the sensor on the simulated wrist of the simulator, then control the micro pressurization system to inflate to 150 mmHg, turn off the integrated air pump, and keep it for a while. Finally, record the air pressure value in the micro pressurization system measured by the air pressure sensor to verify the micro pressurization system airtightness. Repeat the above experiment five times. As shown in Figure 8, within the 40 s after the integrated air pump is turned off, the air pressure can remain above 96.5% of the closed air pressure, which means that the airtightness of the system is excellent.

### 3.3. Consistency of Different Positioning of the Sensor

**Figure 9:**
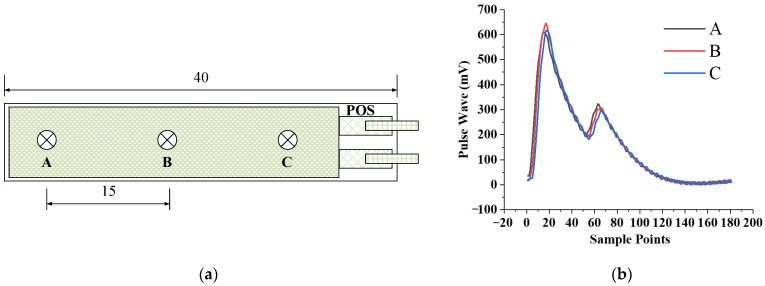
Consistency test of different positioning of the sensor: (**a**) The positions of the three points A, B, and C; (**b**) Pulse wave measured at three points A, B, C.

To verify the consistency of different positionings of the sensor, we choose three positions A, B, and C on the sensor, whose positions are shown in Figure 9. Among them point B is the center position of the sensitive area of the sensor. After the assembly is completed, press the three points A, B, and C of the sensor on the test device, respectively, to collect pulse wave information at the three positions. The above experiment was repeated three times, and the average amplitudes of the pulse wave at the three positions were shown in the Figure 9.

The experimental results show that the pulse wave amplitude collected at point B is the largest, the pulse wave amplitude collected at point A is 4.76% smaller than that of point B, and the amplitude of pulse wave collected at point C is 4.19% smaller than that of point B, which are all less than 5%. Therefore, different positioning of the sensor has little influence on measuring results. In the following experiments, unless otherwise specified, we collect pulse waves at the position point B of the sensor, which can not only obtain the maximum signal amplitude, but also help to cover a sufficient range to ensure that the pulse wave can be monitored.

### 3.4. Repeatability of Flexible Compound Sensor

To verify the repeatability of the applied variable pressure of the micro pressurization system, we performed a pressure test on the flexible compound sensor. The sensor is worn on the simulated wrist of the simulator. Control the micro pressurization system to inflate to 150 mmHg and record the change in the value of the air pressure sensor during the entire pressurization process. Repeat the above experiment five times.

To evaluate the repeatability of the flexible compound sensor, which also reveals the reliability and stability of the sensor, the equation for calculating the repeatability of the sensor is shown as follows:

(1) $$s_{i}{}^{2}=\frac{1}{m-1}\overset{m}{\underset{j=1}{\sum }}{\left(y_{ij}-{\bar{y}}_{i}\right)}^{2}$$

(2) $$s=\sqrt{\frac{1}{2n}\overset{n}{\underset{i=1}{\sum }}s_{i}{}^{2}}$$

where y is the measured value, $s_{i}$ is the variance of the measurement point i, m represents the number of repetitions of test experiment, in this experiment, the value is five, n is the number of measurement points.

**Figure 10:**
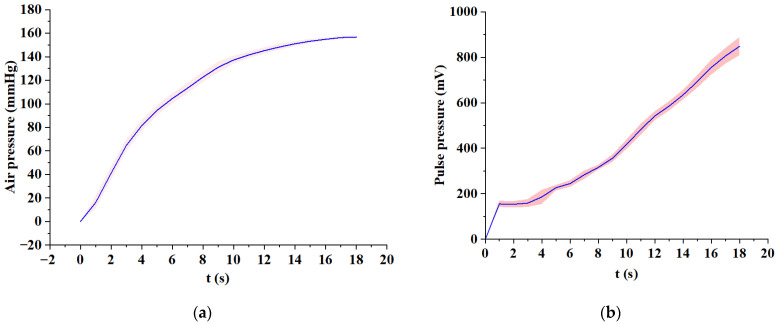
Repeatability test of the flexible compound sensor. Mean and standard deviation of air pressure (**a**) and pulse pressures (**b**) under 0–150 mmHg.

When the micro pressurization system applies continuous pressure to the sensor, the pulse pressure signal and the applied static pressure signal are recorded and used to evaluate the repeatability of the composite pressure sensor. The curve drawn by repeating the above process is shown in Figure 10. The standard deviation of each measurement point is calculated according to Equations (1) and (2). It can be seen from the Figure 10 that the air pressure value and the pulse pressure value under continuously varying pressure show a great linear correlation and stable standard deviation. To check the standard deviation more specifically, we sample the standard deviation every 1 s, and the relative standard deviation values of approximately 18 s are shown in Table 2.

As shown in Table 2, the relative standard deviation of pulse pressure and the relative standard deviation of air pressure in the low-pressure stage (1 s to 4 s) is greater than in the medium pressure and high-pressure stages, and the relative standard deviation values are also unstable. This phenomenon is caused by the fact that the wristband is less inflated in the initial stage and the sensor is not completely in close contact with the test equipment. With the gradual increase of airbag pressure, the relative standard deviations of static pressure and dynamic pressure in the middle-pressure section and the high-pressure section gradually decrease and become stable. The relative standard deviation of static pressure is reduced from 5.6% to 1.5% in high-pressure sections, showing good stability. The relative standard deviation of pulse pressure produced large fluctuations during the entire compression process and the value was much larger than the relative standard deviation of static pressure. However, the relative standard deviation of the pulse pressure was always kept in a low range (from 16.7% Reduced to 3.4%), which will not have a major impact on data analysis. Therefore, during the entire pressurization process, the relative standard deviation of the pulse pressure and the air pressure is kept at a low value, which proves that the pulse pressure sensor and the barometric pressure sensor work stably during the entire process.

### 3.5. Verification of Pulse Pressure and Air Pressure Collection

**Figure 11:**
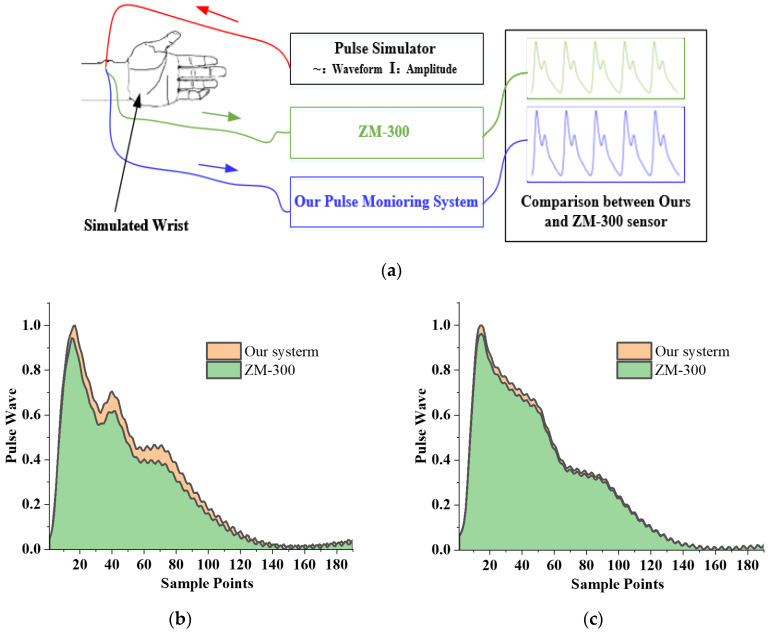

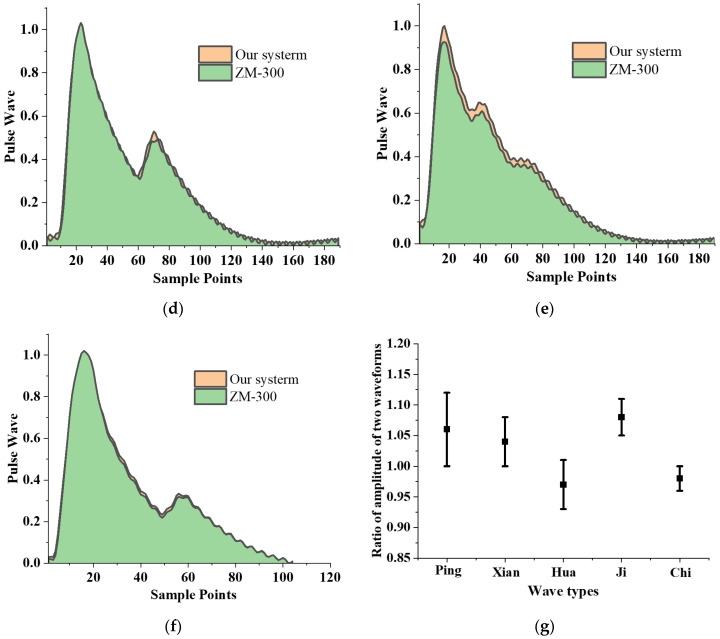
Verification experiment of dynamic characteristics between our sensor and ZM-300 sensor: (**a**) Schematic diagram of the experiment; (**b**–**f**) Result of waveform comparison of five kinds of pulse, Ping, Xian, Hua, Chi, and Ji; (**g**) Result of amplitude comparison of five kinds of pulse, Ping, Xian, Hua, Chi, and Ji.

To evaluate the effectiveness of the flexible compound sensor, we compared the sensor we proposed with the sensor on the ZM-300 [1], which meets TCM’s technical standards for pulse wave detection, and is generally considered to be a standard pulse wave detection system. A verification experiment is designed, as shown in Figure 11. The pulse simulator is used to generate the five most common specific waveforms in TCM to simulate the pulse wave of the human body, and then let our proposed sensor and the ZM-300 sensor measure. The default unit of the output value of the ZM-300 sensor is g/cm^2^, which is converted to kPa to keep the unit consistent with the output value of our sensor. Then, the ratio of the amplitude of the waveform captured by the two sensors is calculated. We have measured five kinds of pulse, namely Ping, Xian, Hua, Ji, and Chi. For each group of pulse waveforms, and we tested them ten times.

The amplitude of the pulse simulator is much larger than the usual pulse wave amplitude, ensuring that the pulse wave can always be recorded without loss. The test results of these five pulse waveforms are shown in Figure 11. From the perspective of waveform similarity, five kind of pulse waveforms were collected with high similarity by our system and ZM-300, respectively. As shown in Table 3, The Pearson correlations between the five kind of pulse waves are 0.99, 0.97, 0.97, 0.98 and 0.99 separately, which means that the pulse wave waveform acquired by our system is very similar to the ZM-300. From the perspective of amplitude, the amplitude ratios of these five kinds of pulse are 1.06, 1.04, 0.97, 1.08 and 0.98 separately, and the average amplitude ratio is 1.03, which shows that the linearity of our flexible compound sensor is only slightly different from that of the ZM-300. The standard deviation of the amplitude ratio of these five types of pulse is 4.00%, which proved that the consistency of the sensor is outstanding. However, our system can automatically collect. Compared with the ZM-300 manual collection, the time required for collection from 5 min is greatly shortened to 30 s.

### 3.6. Verification of Best Pulse Wave Positioning

**Figure 12:**
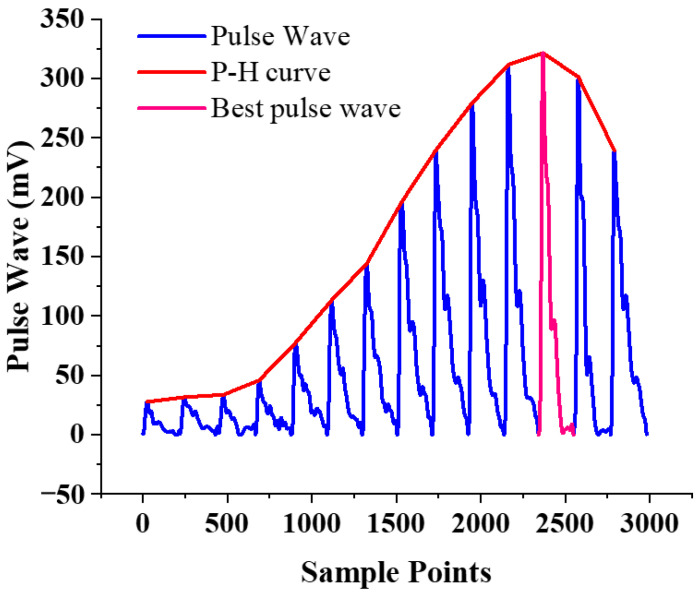
Experimental result of best pulse wave positioning.

To test the performance of best pulse wave positioning of the pulse wave monitoring system we proposed, firstly, the system is worn on the simulated wrist of the simulator. Control the micro pressurization system to inflate, and record the variation in the value of the pulse wave and air pressure during the entire pressurization process. After real-time processing by the algorithm, the experimental result is shown in Figure 12. The system can locate the best pulse wave and corresponding air pressure.

To verify that the system has the same performance for different individuals, 20 volunteers in our institute are recruited for measurement. Each volunteer was asked to sit still for five minutes before the test. Table 4 shows the basic information of the volunteers and the experimental result of best pulse wave positioning. There were 19 of 20 people who could correctly locate the best pulse wave, while one person failed to locate it because the volunteer had vigorous movement during the collection process, which is also our next step: to develop algorithms to eliminate interference from motion artifacts. In summary, the accuracy was 95% in 20 people whose differences are obvious, meaning that the proposed system shows good performance for best pulse wave positioning.

### 3.7. Pulse Wave Continuous Monitoring

**Figure 13:**
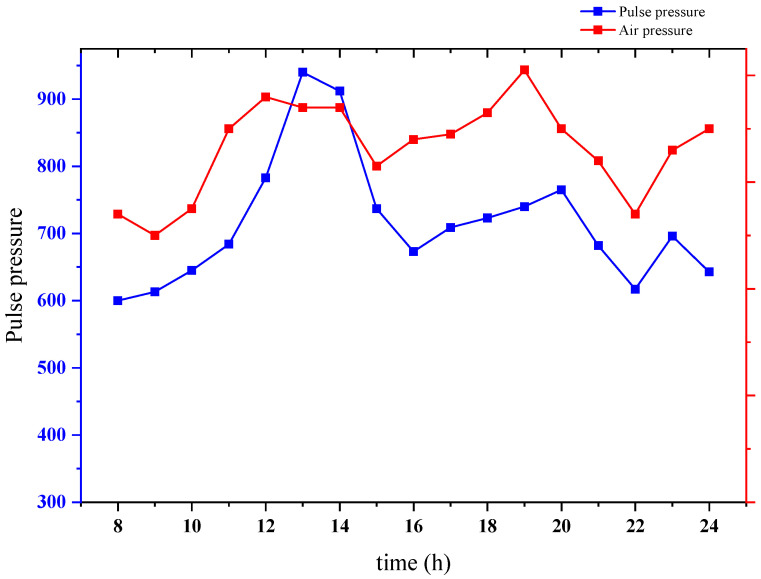
Experimental result of continuous monitoring from 8 a.m. to 12 p.m.

To verify the features for continuous pulse wave monitoring, the pulse wave monitoring system we proposed was worn on a volunteer’s wrist and automatically measured every hour. The main peak of best pulse of the measurement and the corresponding air pressure were calculated in real-time. The experimental result of continuous monitoring from 8 a.m. to 12 p.m. is shown in Figure 13.

According to the experimental results, we can see the varying trend of the main peak of this volunteer’s best pulse wave during the day, which is consistent with the description in TCM. It means that the proposed pulse wave monitoring system based on the flexible compound sensor could indicate the capability of continuous monitoring and reflecting trend differences.

## 4. Conclusions

This study proposed a novel wearable and real-time pulse wave monitoring system based on a flexible compound sensor, which achieved stable acquisition of pulse wave signals under continuously varying pressure. The weight of the whole system is only 52.775 g, the maximum working current is less than 46 mA, and the power consumption of the whole system is less than 160 mW, which meets the requirements of wearable and portable scenarios. Simultaneously, the real-time algorithm we proposed can complete the analysis of the pulse wave and the trend of the best pulse wave peak under continuously varying pressure, which can quickly locate the strongest pulse wave for different individuals. Within 40 s the air tightness can remain above 96.5%. The experiments to verify the reliability, consistency, and effectiveness show that the wearable pulse wave monitoring system can not only realize best pulse wave positioning and pulse wave monitoring under continuously varying pressure, but also indicate the capability of continuous monitoring and reflecting trend differences.

The proposed system and other pulse measurement devices presented in recent studies are summarized in Table 5. Chen [1] and Liu [2] are the previous works used to develop effective pulse wave acquisition devices. Jessica [25] proposed a wearable pulse-taking device. J.C. [24] proposed a wearable pulse acquiring system using airbags. Hsieh [26] proposed a portable pulse tactile recorder system to collect pulse palpation forces and vibrations. Chen J. [27] proposed flexible piezoresistive sensors for pulse monitoring and Li [28] used flexible pressure-sensors to realize the acquisition of arterial pulse signals in the time domain.

In conclusion, the system is feasible for stable acquisition of pulse wave signals under continuously varying pressure, and the analysis of the varying trend of the best pulse wave peak, which is beneficial to pulse diagnosis. Moreover, its small size and low power consumption meet the needs of wearable and portable scenarios, which will play an important role in health monitoring and disease warning. Combining the above advantages, the system has significant research value and commercialization prospects.

In the future, we will improve the function of the system and develop algorithms to eliminate interference from motion artifacts to meet the requirements of exercise monitoring. In addition, we will study the subject in long-term use for users. Furthermore, we will carry out more experiments to make the system more accurate and standardized. This is of important significance to pulse diagnosis and health monitoring.

## 5. Patents

The works presented in this paper are subject to pending China and international patents filed by Institute of Microelectronics of Chinese Academy of Sciences (IMECAS) in China (202110808586.4, 202111553703.3).

## Figures and Tables

**Table 1:** Parameters of PVDF piezoelectric sensor.

Variables	Parameters
Density	1.78 × 10^3^ kg/m^3^
Active area	40 mm × 10 mm
Thickness	28 μm
Capacitance	1.6 nF
Young’s Modulus	2 × 10^9^ N/m^2^
Mylar	5 mil
Sensitivity	14.4 V/N

**Table 2:** Relative standard deviations (RSD) of air pressure and pulse pressures.

Time (s)	1	2	3	4	5	6	7	8	9	10	11	12	13	14	15	16	17	18
RSD of Pulse Pressure	8.7%	9.0%	10.7%	16.7%	5.4%	5.9%	6.4%	3.4%	4.5%	5.0%	5.0%	3.6%	3.5%	3.4%	4.0%	4.2%	5.3%	5.9%
RSD of Air Pressure	33.2%	13.2%	7.8%	5.6%	4.5%	4.1%	3.8%	3.5%	3.5%	2.4%	2.1%	1.9%	1.7%	1.5%	1.1%	0.8%	0.7%	0.8%

**Table 3:** Pearson correlation between the five kind of pulse waves collected by our system and ZM-300.

Pulse Type	Ping	Xian	Hua	Chi	Ji
Pearson correlation	0.99	0.97	0.97	0.98	0.99

**Table 4:** Basic information of 20 volunteers and the experimental result of best pulse wave positioning.

Variables	Parameters	Experimental Result		
		YES	NO	Accuracy
Gender		19	1	95%
Male	10			
Female	10			
Age (year)	29 ± 5.0 (22–46)			
Height (cm)	174.6 ± 7.4 (153–190)			
Weight (Kg)	72.5 ± 18.3 (41–115)			
BMI	22.04 ± 3.4 (16.1–38.9)			

**Table 5:** Comparisons with other pulse measurement systems.

System	Jessica et al. [25]	Liu et al. [2]	J.C. et al. [24]	Chen C. et al. [1]	Hsieh et al. [26]	Chen J. et al. [27]	Li et al. [28]	Proposed
Wearable	YES	NO	YES	NO	YES	YES	YES	YES
Real-time	NO	NO	NO	NO	NO	NO	NO	YES
Weight	Not mentioned	1164.4 g	~800 g	Not mentioned	10 g	Not mentioned	Not mentioned	52.8 g
Portable	NO	NO	NO	NO	YES	YES	NO	YES
Flexible	NO	YES	NO	YES	NO	YES	YES	YES
Pulse-taking pressure acquiring	NO	YES	YES	YES	NO	NO	YES	YES
Measure under continuously changing pressure	NO	NO	YES	NO	NO	NO	NO	YES
Pressurization method	Manually	Pump and air bag	Pump and air bag	Pump and air bag	Manually	Manually	Manually	Integrated air pump (weight: 2.48 g) and air bag
Year of publication	2017	2018	2019	2020	2021	2021	2021	2022

## Data Availability

The raw/processed data required to reproduce these findings cannot be shared at this time as the data also form part of an ongoing study.
